# Factors affecting the isolation and diversity of marine sponge-associated bacteria

**DOI:** 10.1007/s00253-022-11791-8

**Published:** 2022-02-01

**Authors:** Yitayal S. Anteneh, Qi Yang, Melissa H. Brown, Christopher M. M. Franco

**Affiliations:** 1 Medical Biotechnology, College of Medicine and Public Health, Flinders University, Bedford Park, SA 5042 Australia; 2 Department of Medical Microbiology, College of Medicine, Addis Ababa University, Addis Ababa, Ethiopia; 3 Centre for Marine Bioproducts Development, College of Medicine and Public Health, Flinders University, Bedford Park, SA 5042 Australia; 4 Center for Marine Drugs, State Key Laboratory of Oncogene and Related Genes, Department of Pharmacy, School of Medicine, Renji Hospital, Shanghai Jiao Tong University, Shanghai, 200127 China; 5 College of Science and Engineering, Flinders University, Bedford Park, SA 5042 Australia

**Keywords:** Bacterial diversity, Marine sponges, South Australia, Oxygen levels

## Abstract

**Abstract:**

Marine sponges are an ideal source for isolating as yet undiscovered microorganisms with some sponges having about 50% of their biomass composed of microbial symbionts. This study used a variety of approaches to investigate the culturable diversity of the sponge-associated bacterial community from samples collected from the South Australian marine environment. Twelve sponge samples were selected from two sites and their bacterial population cultivated using seven different agar media at two temperatures and three oxygen levels over 3 months. These isolates were identified using microscopic, macroscopic, and 16S rRNA gene analysis. A total of 1234 bacterial colonies were isolated which consisted of four phyla: *Actinobacteria*, *Firmicutes*, *Proteobacteria*, and *Bacteroidetes*, containing 21 genera*.* The diversity of the bacterial population was demonstrated to be influenced by the type of isolation medium, length of the incubation period and temperature, sponge type, and oxygen level. The findings of this study showed that marine sponges of South Australia can yield considerable bacterial culturable diversity if a comprehensive isolation strategy is implemented. Two sponges, with the highest and the lowest diversity of culturable isolates, were examined using next-generation sequencing to better profile the bacterial population. A marked difference in terms of phyla and genera was observed using culture-based and culture-independent approaches. This observed variation displays the importance of utilizing both methods to reflect a more complete picture of the microbial population of marine sponges.

**Key points:**

- *Improved bacterial diversity due to long incubations, 2 temperatures, and 3 oxygen levels.*
- *Isolates identified by morphology, restriction digests, and 16S rRNA gene sequencing.*
- *At least 70% of culturable genera were not revealed by NGS methods.*

**Supplementary Information:**

The online version contains supplementary material available at 10.1007/s00253-022-11791-8.

## Introduction

Studies on biologically active metabolites are increasingly important as they can be used in multiple biotechnological applications, including as new antibiotics that are effective against multidrug-resistant pathogens. Natural environments are still an important source for the discovery of novel antibiotics (Bull and Stach 2007; Claverias et al. 2015). Metabolite-producing microorganisms from terrestrial environments have been investigated for many years; therefore, the chance of finding novel products from these sources is diminishing. Thus, it is crucial to expand the search for microorganisms from less explored areas such as marine environments (Claverias et al. 2015; Goodfellow and Fiedler 2010).

Marine sponges which comprise the phylum *Porifera*, are one of the earliest metazoans that exist on earth (~630 million years) (Maloof et al. 2010). They are sedentary filter-feeders, able to pump thousands of liters of water each day (Bell 2008), and some have the ability to absorb dissolved organic matter (de Goeij et al. 2008). Marine sponges have a worldwide distribution and are vital members of underwater communities. Being the most dominant and diverse members of the marine community, their diversity could outnumber the combined species diversity of most organisms in the community (Van Soest et al. 2012). According to the world Porifera database (Van Soest et al. 2021), there are more than 9432 species of sponges spread among 680 genera and four different classes—*Calcarea*, *Demospongiae*, *Hexactinellida*, and *Homoscleromorpha* (Gazave et al. 2010).

The mesohyl part of many sponges is heavily populated by microbial symbionts which consist of bacteria, fungi, viruses, and archaea (Taylor et al. 2007; Webster and Taylor 2012). Depending on the amount of microorganisms harbored within the sponges, they are broadly classified as high and low microbial abundance sponges. In the former group, the microbial mass accounts for about 10^8^–10^10^ bacteria per gram wet weight of sponge (Hentschel et al. 2006), which is many fold higher than the amount that exists in the surrounding seawater (Friedrich et al. 2001); in many cases, the microbial symbionts represent about 40–60% of the sponge biomass (Hill et al. 2006). In the second category, low levels of microorganisms (10^5^–10^6^ bacteria per gram wet weight of sponge) exist, which is comparable to the amount found in natural seawater (Hentschel et al. 2006).

Marine sponges are host to a large diversity of bacteria, with one study reporting 26 bacterial phyla (Lee et al. 2011) or more if using molecular techniques (Yang et al. 2019b). These sponge-associated bacteria, via the production of biologically active secondary metabolites, protect the sponges against the harmful effects of pathogens, competitors, fouling organisms, and predation (Taylor et al. 2007). It has been also established that sponges produce active secondary metabolites that can have antimicrobial and anti-inflammatory properties (Brady et al. 2009; Piel 2009). Sponges are one of the most productive marine producers of unique metabolites, with more than 3500 novel compounds having been described from sponges between 1985 and 2008 (Hu et al. 2011; Mehbub et al. 2014). Structural similarities between compounds produced by sponges and microbial secondary metabolites led to the hypothesis that some metabolites from sponges have originated from the associated microbes (Wang 2006). Furthermore, several studies successfully isolated the same compounds from bacteria as those reported from their sponge hosts (Bewley et al. 1996; König et al. 2006; Stierle et al. 1988). Therefore, these observations indicate that marine sponges can be an ideal source of microorganisms having the potential to produce new antimicrobial agents (Flemer et al. 2012).

Previous studies reported the isolation of bacteria from marine sponges which displayed a variety of biological activities (Hentschel et al. 2001; Hu et al. 2011; Kennedy et al. 2009; Muscholl-Silberhorn et al. 2008; O’Halloran et al. 2011; Radjasa et al. 2011). South Australian marine environments are a source of many sponge species with approximately 60% of them being endemic (Sorokin and Currie 2008). Variations in the chemical composition among sponges in South Australia have been reported (Sorokin et al. 2007; Sorokin and Currie 2008), and it is expected that the associated bacteria could also be different either in terms of diversity or in the type of metabolites they produce (Anteneh et al. 2021). Therefore, this study was designed to isolate bacteria from marine sponges, investigate factors affecting their relative diversity, and compare the diversity with a culture-independent approach. It was decided that the isolation would utilize differences in medium composition, plating larger volumes of samples, and incubation for lengthy periods under different temperatures and aerobic conditions. This isolation strategy could be compared to the more elaborate use of growth chambers that allow diffusion of sponge metabolites (Steinert et al. 2014), or co-cultivation of sponge samples (Knobloch et al. 2019), and in situ enrichment (Jung et al. 2021) prior to plating.

## Materials and methods

### Sponge sample collection, processing, and classification

Twelve sponge samples were collected from two South Australian marine environments by scuba diving. Specifically, four samples coded as GB 1, GB 08, GB 21 and GB 23 were collected from Glenelg Blocks (34° 58′ 406′′ S, 138° 30′ 494′′ E), and eight sponge samples coded as RB 1, RB 2, RB 3, RB 11, RB 12, RB 16, RB 17, and RB 18 were collected from Rapid Bay Jetty (35° 52′ 29′′ S, 138° 18′ 54′′ E), at a depth of 10–15 m, water temperature of 15°C, and salinity of 36.5–37 PSU. Glenelg Blocks is a city beach where human and domestic animal contacts are high, and there is a local river containing road and housing runoff. In contrast, Rapid Bay Jetty has a much lower frequency of human contact. Sponges were sampled under Exemption Permit Number 9902620 by the South Australian Research Development Institute (SARDI), issued by Primary Industries and Regions South Australia.

The samples were collected in Ziploc plastic bags containing fresh seawater and transported to the laboratory in an icebox. In the laboratory, the survival of the sponge samples was maintained, using an aquarium system for up to 5 days. The sponge samples were processed as described previously in similar studies (Abdelmohsen et al. 2010; Kuo et al. 2019). Briefly, the sponges were thoroughly rinsed with 2 l of autoclaved natural seawater, and the surface of the sample disinfected with 70% ethanol. This was followed by drying of the samples in a sterile laminar flow chamber, and a part removed and processed immediately for microbial isolation, while some parts were kept in a jar with 70% ethanol for sponge morphological identification, and the rest was frozen (−80°C) in sterile Ziploc bags for future analysis.

Seven sponge samples were classified by morphological characterization as described previously (Hooper and van Soest 2002). Histological sections and spicule preparation followed the methods in “Sponguide” (Hooper 2000). All classifications were in line with the revised Demosponge classification (Morrow and Cardenas 2015).

The remaining five sponge samples collected from Rapid Bay (RB 11, RB 12, RB 16, RB 17, and RB 18) were analyzed using 28S rRNA gene sequencing. Sponge DNA from frozen samples were extracted by a conventional hexadecyltrimethylammonium bromide (CTAB)-based protocol (Taylor et al. 2004). In short, the frozen sponge samples were crushed in liquid nitrogen and their tissue lysed with CTAB extraction buffer. The mix was then combined with polyvinylpyrrolidone (PVP) and β-mercaptoethanol to remove tannins and phenolic compounds. The addition of phenol:chloroform:isoamyl allowed the separation of nucleic acids from proteins and polysaccharides, and finally the DNA was precipitated with cold isopropanol and reconstituted with 50-μl injection water and stored at −20°C. The quality and the quantity of the DNA were assessed with a Nanodrop 1000 Spectrophotometer (Thermo Scientific, Wilmington, DE, USA) and those with high quality were considered for PCR reactions.

The DNA samples were amplified, sequenced, and analyzed following previous methods (Yang et al. 2017). Briefly, D3-D5 regions of the 28S rRNA gene of sponge DNA were amplified by the primer set NL4F (5′-GAC CCG AAA GAT GGT GAA CTA-3′,) and NL4R (5′-ACC TTG GAG ACC TGA TGC G-3′) (Nichols 2005). Thermocycler conditions were as follows: a 10-min initial denaturation at 95 °C; 35 cycles of 95 °C for 1 min, 56 °C for 1 min, and 72 °C for 1 min; and a final extension step at 72 °C for 7 min. The PCR products were cleaned and sent for sequencing to Macrogen, South Korea. The sampled sponges are displayed in Figure S1.

### Isolation of sponge-associated bacteria

Approximately 1 cm^3^ of dried sponge pieces were removed from the internal mesohyl area using a sterile scalpel and homogenized using a clean, sterile pestle and mortar. To facilitate homogenization, 3 ml of autoclaved seawater was added. From the homogenate, a tenfold dilution series (10^−1^ to 10^−6^) was prepared, and 100 μl of the three highest dilutions was inoculated in six replicates onto seven isolation media prepared in sterilized natural seawater: Soluble starch yeast extract peptone agar (SYP) (Mincer et al. 2005), asparagine peptone agar (ASP) (Zhang et al. 2006), natural seawater agar (SWA), humic acid-vitamin agar (HV) (Hayakawa and Nonomura 1987), nutrient agar (NA), marine agar (MA) (Mincer et al. 2005), and tryptone soya agar (TSA).

For each incubation type, each sponge sample was plated on seven media, with six replicates and three dilutions. Each set of 126 plates was incubated at 27°C aerobic, 27°C anaerobic, 27°C microaerophilic, and 15°C aerobic conditions, bringing the total to 504 plates per sponge sample. The plates were sealed with parafilm and kept in plastic lunch boxes lined with wet paper towels to prevent drying of the media. The plates were incubated for up to 16 weeks. The anaerobic environment was generated in a sealed jar using an anaerobic generation kit (AnaeroGen^TM^, Sigma-Aldrich), and its adequate establishment was checked using a methylene blue indicator, which turns colorless in the absence of oxygen. Microaerophilic conditions were established by lighting a candle in a candle jar with the lid closed to deplete the oxygen and establish some degree of CO_2_. The appearance of colonies was observed and counted weekly on all plates. Colonies were removed completely and recorded every week. Subculturing and subsequent identification of anaerobic isolates were processed in anaerobic chambers, and at all times the anaerobic environment was maintained within the sealed jar. Similarly, the microaerophilic environment was always maintained during bacterial identification from this incubation condition.

### Purification of isolates

Colonies were picked from primary isolation media once a week, for 12 weeks, as previously described by Kaewkla and Franco (2013) and subcultured onto SYP and NA plates to produce single pure colonies. Pure cultures were stored on plates and agar slants for short periods, and colonies were also placed in sterile 30% glycerol and stored at −80°C for future use (Laich et al. 2013).

### Preliminary identification and categorization of pure isolates

All pure isolates were sub-cultured in a grid system onto SYP, TSA, NA, and MA media and incubated until good growth was achieved. The colony morphology, texture, color, consistency, nature of the spores, and growth pattern on the media were the features employed, if present, for grouping the isolates. Microscopic observation of hyphae in a wet and stained smear and Gram stain characterization of the isolates were also employed to place the isolates into representative clusters.

### Characterization by RFLP of 16S rRNA gene amplicons

Bacterial DNA was extracted by a cetyltrimethylammonium bromide (CTAB) method as described previously (Kurtzman and Robnett 1998). The primers used for the PCR reactions were universal 16S rRNA primers. The 16S rRNA gene was amplified separately in two segments using the primer pairs 27F (5′GAGAGTTTGATCCTGGCTCAG3′) and 765R (5′CTGTTTGCTCCCCACGCTTTC3′) (Bianciotto et al. 1996), which produce a fragment of 738 bp and the pair 704F (5′GTAGCGGTGAAATGCGTAGA3) (Bianciotto et al. 1996) and 1492R (5′CACGGATCCTACGGGTACCTTGTTACGACTT3′) (Weisburg et al. 1991), which produce a 790 bp PCR product. All amplification reactions were carried out in a Swift Thermal Cycler (Esco GB Ltd) with reaction cycle of 95°C for 10 min, 35 cycles of 94°C for 1 min, 52°C for 1 min, and 72°C for 2 min, followed by a cycle of 72°C for 10 min and 12°C cooling. The products were analyzed by electrophoresis on 1% (w/v) agarose gel. Restriction fragment length polymorphisms (RFLP) analysis of the amplified product was used to group the isolates according to the method of Cook and Meyers (2003). The amplified products using the primers 27F and 765R were digested first with *Hha*I and in some cases with *Pst*I restriction enzymes for RFLP-based categorization of the isolates. Representative PCR products from each RFLP pattern were sent for sequencing to Macrogen, South Korea, and the results were subjected to a BLASTN search of the NCBI 16S rRNA database.

### Temperature and NaCl tolerance tests

For temperature tolerance tests, all bacterial isolates were inoculated on ASP and SYP agars in a grid of four and incubated at 3, 15, 27, 37 and 45°C for 2 weeks. In the same manner, the bacterial isolates were inoculated onto ASP and SYP media containing NaCl concentrations (w/v) of 0, 1, 2, 3, 4, 6, 8, 10, 12, 14, and 16% and incubated at 15°C and 27°C for 2 weeks. Both tolerance tests were carried out in duplicate on both media. The agar plates for NaCl tolerance were prepared using distilled water to remove the effect of any unintended salt. Results were recorded as positive or negative for all treatments by observing the presence or absence of colony growth at specified incubation periods.

### Sponge metagenomic DNA isolation, sequencing, and data processing

The sponge species with the highest (*Aplysilla sulfurea*) and lowest (*Carteriospongia foliascens*) culturable bacterial diversity were selected to conduct amplicon-based metagenomic sequencing on the next-generation sequencing platform Illumina MiSeq. DNA was extracted (Yang et al. 2015), and the purity and quantity were determined with a NanoDrop ND-1000 spectrophotometer (Thermo Scientific, Wilmington, DE, USA). The DNA samples (A260/280: 1.8–2.0; Conc. > 100 ng/μl) extracted from different sponge individuals for each species were selected and kept at −20 °C for subsequent sequencing.

Illumina MiSeq amplicon library was prepared as previously described (Yang et al. 2019a). Briefly, the three primers for the selected 16S rRNA gene region V1V3 were 28F-519R (28F: 5′-GAGTTTGATCNTGGCTCA G-3′; 519R: 5′-GTNTTACNGCGGCKGCTG-3′) (Croué et al. 2013), 518F-926R for the V4V5 region (518F: 5′-CCAGCAGCYGCGGTAAN-3′; 926R: 5′-CCGTCA ATTCNTTTRAGT-3′) (Nelson et al. 2014), and 803F-1392R for the V5V8 region (803F: 5′-TTAGANACCCNNGTA GTC-3′; 1392R: 5′-ACGGGCGGTGWGTRC-3′) (Engelbrektson et al. 2010). PCR was performed as previously described (Caporaso et al. 2011). The reaction conditions were 2 mM MgCl2, 0.2 μM each primer, and 200 μM dNTPs. The PCR conditions were 94°C for 3 min, followed by 94°C for 45 s, 50°C for 60 s, and 72°C for 90 s in 35 cycles, and a final elongation step at 72°C for 10 min.

Sequencing was run multiple times (*n* = 3) for each amplicon. The demultiplexing and quality filter (at Phred >  = Q20) for the Illumina MiSeq dataset was processed by script split libraries.py in QIIME pipeline (version 1.9.1) (Caporaso et al. 2010). The multiplexed reads were assigned to samples based on their nucleotide barcode (demultiplexing). Quality filtering was performed based on the characteristics of each sequence, removing any low quality or ambiguous reads. Closed-reference picking was selected in our study (see OTU picking strategies in QIIME) (Caporaso et al. 2010). By default, QIIME applied the uclust (Edgar 2010) consensus taxonomy classifier to attempt to assign taxonomy to each representative sequence. The OTU representative sequences were aligned using PyNAST tool (Caporaso et al. 2010), and the filtered alignment file was then used to build a phylogenetic tree using a tree-building program FastTree (Price et al. 2010)). Finally, an OTU table (otu table.biom) was summarized to show the OTU abundances with taxonomic identifiers for each OTU based on Greengenes taxonomy. These steps were performed for each primer pairs separately and the OTU tables for each primer set were merged. The raw NGS reads were deposited in the GenBank at the National Center for Biotechnology Information (BioProject ID: PRJNA490791).

## Results

### Characterization of sponge samples

Sponge samples coded as GB for Glenelg Blocks: GB 1 classified as *Geodia* sp.; GB 08, GB 21 and GB 23 were *Chondrosida* spp. The sponge samples from Rapid Bay (RB 1, RB 2, and RB 3) were morphologically identified as *Ircinia* sp., *Poecilosclerida* sp., and *Crella* sp., respectively. NCBI database was used for BLASTn of the sequences obtained, and the five sponge samples were identified as *Sarcotragus* sp. (EF646841) (RB 11), *Carteriospongia foliascens* (KC869574) (RB 12), *Aplysilla sulfurea* (EF646837) (RB 16), *Dendrilla* sp. (KU533858) (RB 17), and *Tedania tubulifera* (KJ620377) (RB 18).

### Bacterial isolation and morphological identification

A total of 1234 colony forming unit (CFU) bacteria were isolated from the many bacteria cultivated from the 12 sponge samples collected from Glenelg (310) and Rapid Bay (924). The bacterial isolates were selected based on different macro- and microscopic features when grown on primary and identification media. The microscopic properties of these bacteria indicated that the majority were Gram-positive (637), followed by filamentous (375), and Gram-negative (222) species. These bacterial isolates were further categorized into 383 groups, based on morphological features after growth on three media, together with their microscopic characteristics, comprising 195 Gram-positive bacteria, 118 bacteria with mycelial filaments, and 70 Gram-negative bacilli and coccobacilli.

### Identification of the genera in PCR–RFLP patterns with HhaI and PstI digestion and 16S rRNA gene sequencing

Partial 16S rRNA gene amplicons of the 383 bacterial isolates were subjected to restriction enzyme digestion first with the restriction enzyme *Hha*I. This enabled categorization of the 383 bacteria into 32 distinct patterns, and their similarities were cross checked by observing the macro and microscopic features of strains within each pattern group. Pattern groups 7, 21 and 31, which had members which varied morphologically, were examined further by a *Pst*I digestion resulting in six more patterns.

As indicated in Table 1, 63 bacteria were selected randomly, with representatives from each RFLP pattern, and subjected to partial 16S rRNA gene sequencing. The result placed the 383 bacteria into 21 genera under the four phyla of *Actinobacteria*, *Firmicutes*, *Proteobacteria*, and *Bacteroidetes*. Of interest, almost all (105 of 111) the *Streptomyces* spp. were isolated aerobically at 27°C with only two actinobacterial genera being isolated anaerobically. In contrast, all except two of the non-actinobacterial genera could be isolated under anaerobic conditions (Table 1).

**Table 1:** Bacteria identification to genus level via PCR–RFLP with *Hha*I and *Pst*I restriction enzyme digestion and 16S rRNA gene sequencing

Pattern	Isolated from				Fragment sizes	Genus	Phylum
	27 °C aerobic	27 °C anaerobic	27 °C microaerophilic	15 °C aerobic			
15	4^c^	-	1	2	600, 500, 200	*Gordonia*	*Actinobacteria*
19	2^c^	-	2^c^	1^c^	400, 380, 350, 250, 180	*Isoptericola*	*Actinobacteria*
1^a^	1^c^	-	-	-	Uncut	*Janibacter*	*Actinobacteria*
21	7	-	3	9^c^	400, 160, 100	*Kocuria*	*Actinobacteria*
33^b^	5^c^	-	-	1	400, 160, 110, 100	*Kocuria*	*Actinobacteria*
34^b^	5^c^	-	-	1	400, 300, 150, 100	*Kocuria*	*Actinobacteria*
20	11	4^c^	2	-	600, 350, 250, 200, 180	*Microbacterium*	*Actinobacteria*
23	2	-	-	1^c^	400, 350, 300	*Micrococcus*	*Actinobacteria*
24	2^c^	-	-	3	500, 250, 240	*Micrococcus*	*Actinobacteria*
26	3^c^	-	-	1	350, 160, 150, 120	*Micrococcus*	*Actinobacteria*
29	1^c^	-	-	2	300, 250, 180	*Micrococcus*	*Actinobacteria*
17	2 ^c^	-	1	-	400, 220, 200, 100	*Mycolicibacterium*	*Actinobacteria*
30	7^c^	-	-	-	400, 320	*Pseudonocardia*	*Actinobacteria*
12	1	3^c^	2	2	350, 310	*Rhodococcus*	*Actinobacteria*
38^b^	3^c^	-	-	-	500, 450, 350, 210, 100	*Streptomyces*	*Actinobacteria*
37^b^	2^c^	-	-	-	350, 310, 250, 200	*Streptomyces*	*Actinobacteria*
31	76 (9)^c^	-	3	1	500, 160, 100	*Streptomyces*	*Actinobacteria*
32	24 (2)^c^	-	2	-	450, 160, 80, 100	*Streptomyces*	*Actinobacteria*
3	3^c^	-	-	-	400, 210	*Muricauda*	*Bacteroidetes*
1^a^	-	1^c^	-	1^c^	Uncut	*Bacillus*	*Firmicutes*
5	2	2	2^c^	2	450, 200	*Bacillus*	*Firmicutes*
7	9 (2)^c^	16^c^	8 (2)^c^	17 (2)^c^	350, 200, 180	*Bacillus*	*Firmicutes*
8	2	1^c^		2	350, 200, 100	*Bacillus*	*Firmicutes*
13	5^c^	5	3^c^	4	350, 250, 200, 100	*Bacillus*	*Firmicutes*
35^b^	2^c^	-	-	1	400, 300, 220, 200	*Bacillus*	*Firmicutes*
36^b^	-	-	-	2^c^	500, 350, 250, 220, 200, 180	*Bacillus*	*Firmicutes*
22	2		1^c^	1	400, 300, 100	*Bacillus*	*Firmicutes*
9	2	3	2^c^	-	350, 220, 180, 150, 100	*Fictibacillus*	*Firmicutes*
10	-	4^c^	2^c^	-	300, 200, 150	*Flasibacillus*	*Firmicutes*
18	5^c^	-	-	-	600, 500, 220, 100	*Staphylococcus*	*Firmicutes*
16	1	2	-	2^c^	700, 120	*Leisingera*	*Proteobacteria*
4	1^c^	2		2	450, 400	*Limimaricola*	*Proteobacteria*
6	3^c^	1	2	6^c^	350, 200, 180	*Limimaricola*	*Proteobacteria*
25	-	1^c^	-	-	420, 400	*Pseudoalteromonas*	*Proteobacteria*
14	1^c^	2	1	2	600, 180, 100	*Pseudomonas*	*Proteobacteria*
11	-	3^c^	-	2	350, 180	*Rhodovulum*	*Proteobacteria*
1^a^	1	-	-	-	Uncut	*Sulfitobacter*	*Proteobacteria*
2	2	7	3^c^	6^c^	400, 350	*Sulfitobacter*	*Proteobacteria*
27	1	3^c^	1	3^c^	400, 350, 300, 220, 200, 100	*Sulfitobacter*	*Proteobacteria*
28	-	1	1^c^	3	600, 200	*Sulfitobacter*	*Proteobacteria*
Total	200	60	43	80			

^a^Different morphotypes separated by sequencing; ^b^Patterns obtained after *Pst*I digestion; ^c^Isolates selected for 16S rRNA gene sequencing. The number of isolates sequenced is indicated in ()

The percentage of 16S rRNA gene sequence similarity with type cultures (Table S1) indicated that 26 bacteria (41.3% of those sequenced) had a sequence similarity of less than 99% and were therefore candidates for full sequencing to determine if they were novel species. Table 2 presents the distribution of bacterial isolates into various phyla and genera. Of the 383 bacteria isolated, the most abundant (53.5%) belonged to the Phylum *Actinobacteria*, followed by *Firmicutes* (28.5%), *Proteobacteria* (17.2%), and *Bacteroidetes* (0.8%). Among the 21 genera identified, the genus *Streptomyces* was the most frequently isolated (30%) followed by *Bacillus* (23.8%) with the least abundant being *Janibacter* and *Pseudoalteromonas*, each with one isolate.

**Table 2:** The abundance of bacterial isolates in terms of phylum and genus

Bacteria isolates	Abundance (%)
*Actinobacteria* * Gordonia* * Isoptericola* * Janibacter* * Kocuria* * Microbacterium* * Micrococcus* * Mycolicibacterium* * Pseudonocardia* * Rhodococcus* * Streptomyces*	205 (53.5) 7 (1.8) 5 (1) 1 (0.3) 31 (8.1) 17 (4.4) 15 (3.9) 3 (0.8) 7 (1.8) 8 (2.1) 111 (30)
*Firmicutes* * Bacillus* * Fictibacillus* * Falsibacillus* * Staphylococcus*	109 (28.5) 91 (23.8) 7 (1.8) 6 (1.6) 5 (1)
*Proteobacteria* * Leisingera* * Limimaricola* * Pseudoalteromona* * Pseudmonas* * Rhodovulum* * Sulfitobacter*	66 (17.2) 5 (1) 17 (4.4) 1 (0.3) 6 (1.6) 5 (1.3) 32 (8.4)
*Bacteroidetes* * Muricauda*	3 (0.8) 3 (0.8)
Total	383 (100)

### Diversity of bacterial isolates at different parameters

**Fig. 1:**
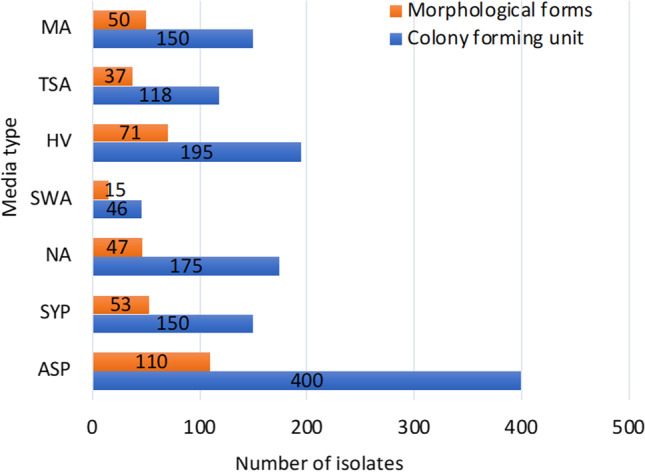
The distribution of CFU (1234) and morphological types (383) of bacterial isolates

The effect of media on bacterial abundance and diversity was examined (Fig. 1 and Table 3). The highest number of CFU and morphological forms were observed on ASP medium followed by HV and with the lowest on the SWA medium (Fig. 1). Among the 21 genera, 16 were recovered using ASP medium, 14 from TSA, 13 from each of SYP, MA, and NA, 12 from HV and the lowest (9) from SWA medium (Table 3). Specifically, all media supported the isolation of *Streptomyces*, *Micrococcus*, *Bacillus*, *Isoptericola* and *Kocuria*, and with the exception of SWA, of *Gordonia*; five media allowed for isolation of *Microbacterium* (except SWA & HV) and *Rhodococcus* (except SAW and MA); four media allowed for isolation of *Leisingera* (except SYP, NA, and TSA), *Fictibacillus* (except ASP, SWA, and HV), *Limimaricola* (except ASP, SWA, and TSA), *Muricauda* (except ASP, SWA, and HV), and *Pseudonocardia* (except TSA, NA, and SYP); three media allowed for isolation of *Flasibacillus* (ASP, NA, and TSA), *Staphylococcus* (ASP, SWA, and TSA), *Sulfitobacteria* (ASP, SWA, and HV) and *Mycolicibacterium* (SYP, NA, TSA); two media allowed for *Pseudomonas* (ASP, TSA), and only a single medium allowed for isolation of *Rhodovulum* (ASP), *Pseudoalteromonas* (SYP), and *Janibacter* (ASP). ASP medium was the most effective both in the number of bacteria isolated as well as genus diversity.

**Table 3:** The distribution of bacterial genera obtained from seven isolation media

Genus	Media type							Total
	ASP	SYP	NA	SWA	HV	TSA	MA	
*Gordonia*	10	4	2	-	1	2	3	22
*Isoptericola*	6	9	4	3	5	2	3	32
*Janibacter*	3	-	-	-	-	-	-	3
*Kocuria*	30	19	11	5	8	15	10	98
*Microbacterium*	11	8	12	-	-	14	9	54
*Micrococcus*	15	9	5	2	3	6	8	48
*Mycolicibacterium*	-	5	2	-	-	3	-	10
*Pseudonocardia*	8	-	-	2	8	-	4	22
*Rhodococcus*	8	6	3	-	4	4	-	25
*Streptomyces*	100	44	43	13	90	17	45	352
*Muricauda*	-	4	1	-	-	3	2	10
*Bacillus*	85	26	56	5	42	32	43	289
*Fictibacillus*	-	-	12	-	-	6	4	22
*Falsibacillus*	6	-	9	-	-	4	-	19
*Staphylococcus*	5	-	-	3	-	8	-	16
*Leisingera*	6	-	-	4	2	-	4	16
*Limimaricola*	-	13	15	-	11	-	15	54
*Pseudoalteromonas*	-	3	-	-	-	-	-	3
*Pseudomonas*	17	-	-	-	-	2	-	19
*Rhodovulum*	16	-	-	-	-	-	-	16
*Sulfitobacter*	74	-	-	9	21	-	-	104
Total	400	150	175	46	195	118	150	1234

**Fig. 2:**
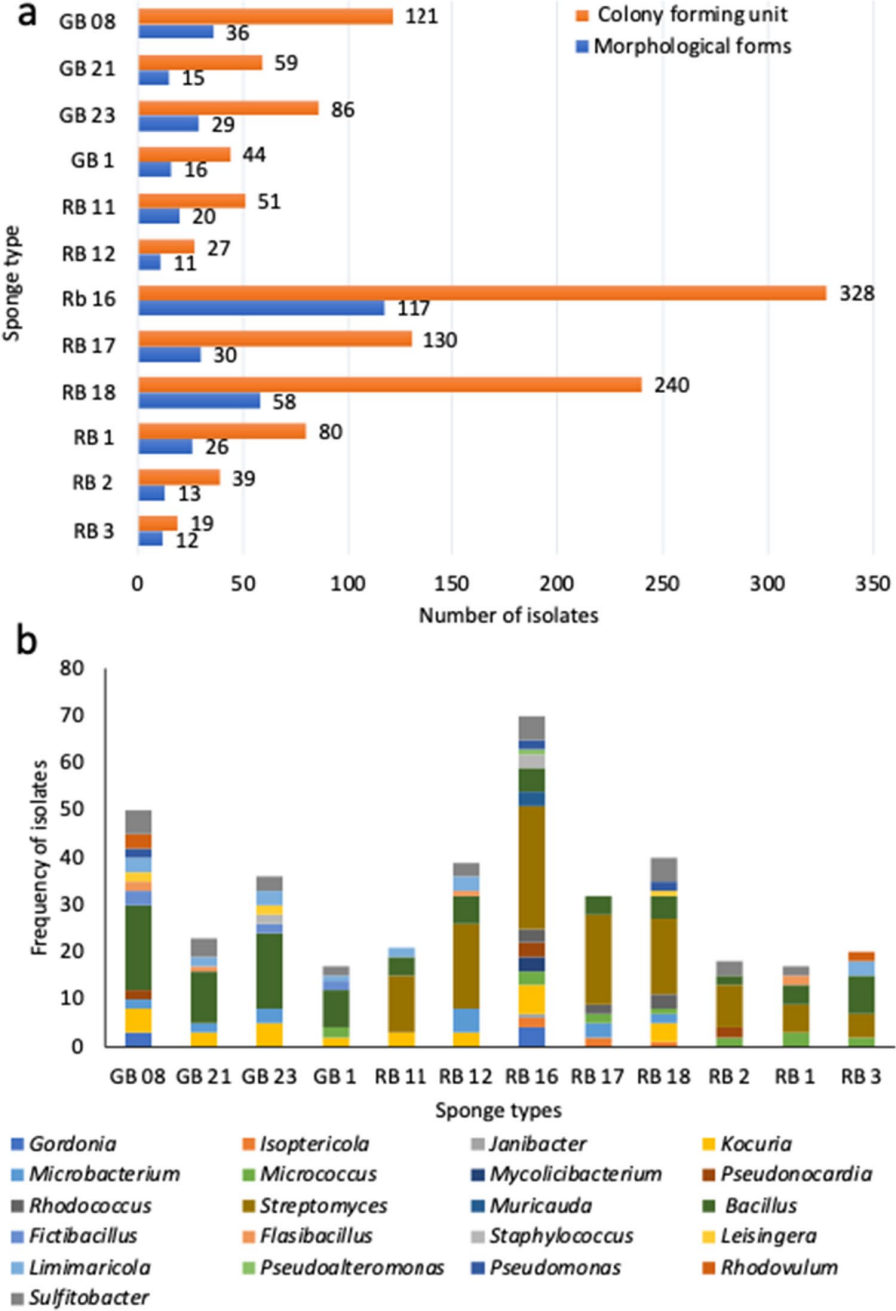
The genus diversity frequency among 12 sponge samples collected from Glenelg and Rapid Bay. **a** Total CFU and morphological forms in each sponge sample. **b** Diversity of bacterial isolates in each sponge sample at the genus level. RB 1 (*Ircinia* sp.), RB 2 (*Poecilosclerida* sp.), RB 3 (*Crella* sp.), RB 11 (*Sarcotragus* sp.), RB 12 (*Carteriospongia foliascens*), RB 16 (*Aplysilla sulfurea*), RB 17 (*Dendrilla* sp.), RB 18 (*Tedania tubulifera*), GB 1 (*Geodia* sp.), GB 08 (*Chondrosida* sp*.*), GB 21 (*Chondrosida* sp*.*), GB 23 (*Chondrosida* sp*.*)

The number and diversity of bacterial isolates were assessed in terms of sponge collection sites and sponge species (Fig. 2). The total bacterial isolates, as well as morphological types, varied considerably among sponge types from the two collection sites. Among the Rapid Bay sponge samples, sample RB 16 (*Aplysilla sulfurea*) yielded the largest number of bacterial isolates (35.5%) and morphological types (41%), followed by sponge sample RB 18 (*Tedania tubulifera*), and sponge RB 17 (*Dendrilla* sp.). Unlike the above samples, lower numbers of bacteria were isolated from the sponges RB 1 (*Ircinia* sp.), RB 2 (*Poecilosclerida* sp.), RB 3 (*Crella* sp.), RB 11 (*Sarcotragus* sp.), and RB 12 (*Carteriospongia foliascens*).

Similarly, among sponge samples collected from the Glenelg blocks, sponge sample GB 08 (*Chondrosida* sp*.*) produced the highest bacterial population (39%) and morphological types (37.5%), followed by GB 23 (*Chondrosida* sp*.*) and GB 21 (*Chondrosida* sp*.*), with the lowest number isolated from GB 1 (*Geodia* sp.) (Fig. 2a).

The distribution of genera also varied within sponge types (Fig. 2b) and among the sites of sponge collection (Table 4). Fourteen different genera (*Gordonia*, *Kocuria*, *Microbacterium*, *Pseudonocardia*, *Bacillus*, *Fictibacillus*, *Falsibacillus*, *Leisingera*, *Limimaricola*, *Pseudomonas*, *Rhodovulum*, *Sulfitobacter*, *Staphylococcus* and *Micrococcus*) were isolated from the four Glenelg blocks sponge samples. Except for *Staphylococcus* and *Micrococcus*, all were isolated from sponge GB 08. Eight genera were isolated from sample GB 23 and six genera from each of sponge sample GB 1 and GB 21. *Falsibacillus*, *Staphylococcus*, *Leisingera* and *Micrococcus* were detected only from Gb 21, GB 23, and GB 1, respectively.

**Table 4:** The diversity of bacterial genera among sites of sponge collection

Genera	Sponge collection sites		Genera	Sponge collection sites	
	Glenelg Blocks	Rapid Bay		Glenelg Blocks	Rapid Bay
*Gordonia*	3	4	*Bacillus*	53	38
*Isoptericola*	0	5	*Fictibacillus*	7	0
*Janibacter*	0	1	*Falsibacillus*	2	4
*Kocuria*	15	16	*Staphylococcus*	2	3
*Microbacterium*	7	10	*Leisingera*	4	1
*Micrococcus*	2	13	*Limimaricola*	9	8
*Mycolicibacterium*	0	3	*Pseudoalteromonas*	0	1
*Pseudonocardia*	2	5	*Pseudomonas*	2	4
*Rhodococcus*	0	8	*Rhodovulum*	3	2
*Streptomyces*	0	111	*Sulfitobacter*	13	19
*Muricauda*	0	3			

Except for the genus *Fictibacillus*, all of the isolated genera were observed from at least one of the eight sponge samples collected from Rapid Bay. Sponge number RB 16 presented 15 genera with the next abundant being RB18 with 10 genera. Except for *Streptomyces* and *Bacillus*, no single genus was shared by all the sponges at this site.

Among 14 genera isolated from the Glenelg Block, 35.7% belong to phylum *Actinobacteria* (*Kocuria*, *Gordonia*, *Microbacterium*, *Micrococcus*, and *Pseudonocardia*), while 50% of the genera isolated from Rapid Bay belong to phylum *Actinobacteria* which included the five genera from Glenelg plus *Streptomyces*, *Isoptericola*, *Janibacter*, *Mycolicibacterium* and *Rhodococcus*. Among non-actinobacterial genera all except *Fictibacillus*, *Pseudoalteromonas* and *Muricauda* were isolated from the two sites, whereas the first was only isolated from Glenelg and the last two only from Rapid Bay.

The abundance and diversity of bacteria at different incubation periods were assessed (Table 5 and Table S2). The cumulative bacterial number sharply increased in the first 3 weeks of incubation, and the rate of change remained constant until 6 weeks followed by a surge until 9 weeks (Figure S2). In terms of diversity at the genus level, in the first 3 weeks of incubation, the isolates were dominated by genera other than from phylum *Actinobacteria*. In the first 3 weeks of incubation, all genera under phylum *Firmicutes* (*Bacillus*, *Fictibacillus*, *Falsibacillus*, and *Staphylococcus*) were isolated but only one genus from phylum *Actinobacteria* (*Streptomyces*) was observed. By week 5, half of the actinobacterial genera and all the genera in the other three phyla were isolated. As the incubation period approached 9 weeks, all the genera were detected, and no new genera were detected until 16 weeks of incubation.

**Table 5:** Bacterial genera diversity isolated with increasing lengths of incubation time

Incubation periods	Genera identified		
< 3 weeks	*Bacillus* *Fictibacillus* *Falsibacillus* *Leisingera*	*Limimaricola* *Muricauda* *Pseudomonas* *Rhodovulum*	*Staphylococcus* *Streptomyces*
4–5 weeks	*Gordonia* *Isoptericola* *Kocuria* *Leisingera*	*Micrococcus* *Microbacetrium* *Pseudoalteromonas* *Sulfitobacter* *Streptomyces*	
6–9 weeks	*Gordonia* *Isoptericola* *Janibacter* *Kocuria* *Limimaricola*	*Microbacterium* *Micrococcus* *Muricauda* *Mycolicibacterium* *Pseudonocardia*	*Rhodococcus* *Streptomyces* *Sulfitobacter* *Rhodovulum*
> 10 weeks	*Streptomyces* *Kocuria* *Microbacterium*	*Limimaricola* *Sulfitobacter* *Pseudonocardia*	

The diversity of the isolated bacteria was assessed at different incubation conditions as indicated in Figure S3. About half of the morphological forms (52.2%) were isolated using 27°C aerobic incubation, followed by 15°C aerobic (20.9%), 27°C anaerobic (15.7%), and 27°C microaerophilic (11.2%) incubations (Figure S3a). In terms of genus diversity, 19 genera, apart from *Falsibacillus* and *Pseudoalteromonas*, were isolated under aerobic conditions, 13 from microaerophilic and 11 genera from anaerobic incubation (Figure S3b). Seven genera were isolated from all oxygen levels; these were *Bacillus*, *Fictibacillus*, *Lamimaricola*, *Rhodococcus*, *Microbacterium*, *Pseudomonas*, and *Sulfitobacter*, while *Pseudonocardia*, *Janibacter*, *Micrococcus*, *Muricauda* and *Staphylococcus* were only isolated under aerobic conditions and *Pseudoalteromonas* isolated only under anaerobic conditions. The isolates were tested for their strict oxygen requirement(s), and the results indicated all of the 60 strains which were isolated from anaerobic incubation grew in aerobic setups, indicative of facultative anaerobic properties, and 50 out of 280 bacteria isolated under aerobic conditions did not grow under strictly anaerobic conditions. All genera were observed at 27°C incubation versus 12 genera (6 of them were from the phylum *Actinobacteria*) at 15°C (Table S2).

### Tolerance tests

**Fig. 3:**
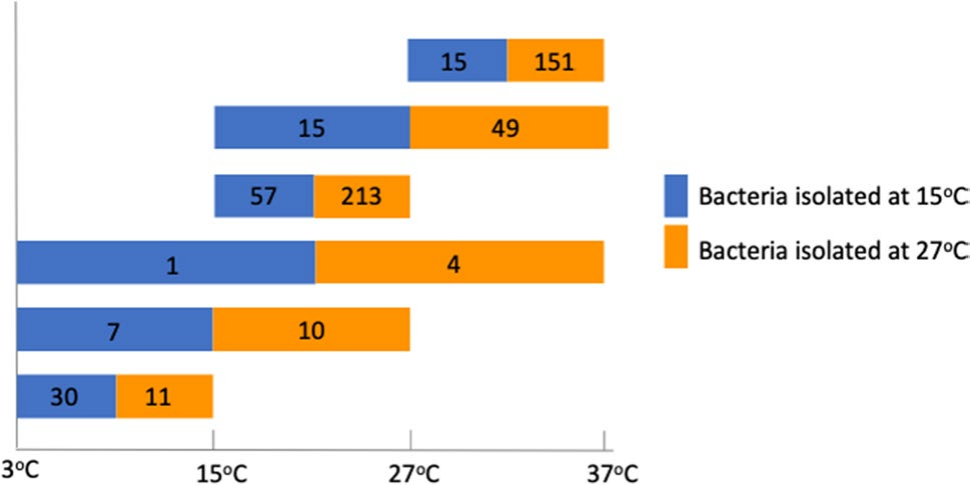
Growth capability at 3 °C, 15 °C, 27 °C, and 37 °C of the 383 bacteria isolated at 15 °C or 27 °C. The numbers in the bar indicate the bacterial strains cultivated in the temperature range (minimum to maximum labelled in *x*-axis); the blue bars represent the bacteria originally isolated at 15 °C, and the orange bars represent the bacteria originally isolated at 27 °C

All 383 bacterial isolates were tested for their tolerance to salt and temperature. Five bacterial isolates were observed to grow at temperatures ranging from 3 to 37°C, with four of them belonging to the genus *Bacillus* and which were initially isolated at 27°C and one from the genus *Pseudomonas*, which was primarily isolated from 15°C incubation (Fig. 3). Twenty-one percent of the isolates had broader tolerances of either 3–27°C or 15–37°C, while the majority of the isolates grew within a narrower temperature range. Bacterial isolates from phylum *Firmicutes* tolerate the above-mentioned wider temperatures (29%) compared to bacterial isolates from phylum *Actinobacteria* (18%), *Proteobacteria* (13%), and none for *Bacteroidetes*. The maximum number of bacterial isolates grew at temperatures of 15–27°C (270) followed by 151 bacteria at a range of 27–37°C and 41 bacteria at a range of 3–15°C. The temperature ranges of 15–27°C were tolerated by 90% of bacterial isolates from phylum *Proteobacteria*, 74% from *Firmicutes*, and 61% from *Actinobacteria*.

The NaCl tolerance tests indicated some bacteria were able to grow in concentrations of 1–14% NaCl (w/v), but the maximum number of bacterial isolates was observed at 4% NaCl. The tolerance declined from 6% and none grew at 16% NaCl. Though a high percentage of isolates (58.7%) did not show a mandatory requirement for any NaCl in the growth medium, a considerable number of bacteria (41.3%) required the presence of NaCl (Figure S4). Specifically, all bacterial isolates from phylum *Bacteroidetes*, 57.6% from *Proteobacteria*, 54% from *Firmicutes*, and 29.8% from *Actinobacteria* required NaCl for their growth. Furthermore, two-thirds of these NaCl-requiring bacteria showed growth at NaCl concentrations of >10%, while it was only 7% for those that could grow in the absence of NaCl. It was also observed that the bacteria grew better in media prepared with seawater compared to media containing NaCl, and the sporulation of some bacteria was enhanced in the presence of a certain concentration of NaCl (data not shown).

### Sponge *Aplysilla sulfurea* and *Carteriospongia foliascens* microbiomes revealed by NGS

Next-generation sequencing was performed on the sponge that showed the highest and lowest genus diversity on cultivation. A total of 12 and 13 microbial phyla were uncovered from *Aplysilla sulfurea* and *Carteriospongia foliascens*, respectively (Table 6), and the complete list of phyla is presented in Table S3. The NGS data also showed a total of 149 and 192 confirmed and candidate genera from *A. sulfurea* and *C. foliascens*, respectively. The complete list of the genera is presented in Tables S4 and S5.

**Table 6:** Microbial phyla and genera revealed for sponges *Aplysilla sulfurea* and *Carteriospongia foliascens* by NGS compared to cultivation

	RB 16 (*Aplysilla sulfurea*)			RB 12 (*Carteriospongia foliascens*)		
	NGS	Isolates		NGS	Isolates	
	Total	Within NGS	Not in NGS	Total	Within NGS	Not in NGS
Phylum	12	4	0	13	3	0
Known genera	137	6	9	183	2	5
Candidate genera	12	0	0	9	0	0

Less than 5% of the genera identified by NGS were able to be isolated. Forty percent of those genera isolated by culture methods (*Pseudoalteromonas*, *Pseudomonas*, *Pseudonocardia*, *Pseudoalteromonas*, *Staphylococcus*, and *Streptomyces*) were shared by NGS*.* However, ten genera out of 16 were not observed by NGS.

## Discussion

The marine environment covers about 70% of the earth’s surface, and its oldest extant animals, marine sponges, are valuable sources of microorganisms (Brinkmann et al. 2017). The marine environments of South Australia contain large numbers of sponge species and most of them are endemic (Sorokin et al. 2007; Sorokin and Currie 2008). However, few studies have reported the isolation and diversity of bacteria from marine sponges from these environments. In this study, the bacterial communities of twelve sponge species were investigated by culturing onto seven different primary isolation media. In comparison, similar studies from Brazil with a comparable number of sponge samples yielded a lower number of 158 CFU (Santos et al. 2010). Several studies reported a number of bacteria from marine sponges with different morphological forms (Bibi et al. 2018; Lafi et al. 2005; Margassery et al. 2012). and our study further supports the finding that marine sponges are a font of bacteria with various microscopic and morphological forms.

When attempting to characterize large numbers of isolates, restriction enzyme digestion of the 16S rRNA gene amplicons followed by sequencing of selected strains was found to be an efficient strategy for their rapid identification (Gernert et al. 2005; Zhang et al. 2006). A combination of two enzymes, *Hha*I and *Pst*I*,* allowed the categorization of the 383 bacteria into 38 manageable groups, which confirmed the high discriminatory power of the two enzymes. The microscopic and morphological forms of each pattern were checked for agreement and whether all bacteria within the same pattern possessed similar properties. Partial 16S rRNA gene sequencing placed the isolates into 21 genera belonging to four phyla, *Actinobacteria*, *Firmicutes*, *Proteobacteria*, and *Bacteroidetes*, commonly reported in most culture-dependent bacterial community studies from marine sponges (Flemer et al. 2012; Graça et al. 2015; Margassery et al. 2012). In agreement with our report, a high abundance of phylum *Actinobacteria* was reported in similar studies (Kennedy et al. 2009; Kuo et al. 2019; Webster et al. 2001). However, other studies reported lower levels of the phylum *Actinobacteria* compared to *Proteobacteria* (Flemer et al. 2012; Graça et al. 2015; Margassery et al. 2012).

Direct plating methods used in our studies gave a large number of isolates but none that could be classified as novel genera defined as having < 97% 16S rRNA gene sequence similarity to known species. In comparison, three other groups used growth chambers placed inside or next to sponge tissue prior to cultivation by plating on up to 5 media. They attempted to isolate previously uncultured bacteria using these methods with some success. Diffusion growth chambers yielded 17% of isolates which were novel (Steinert et al. 2014). Knobloch et al (2019) attempted an ex-situ approach using co-cultivation with sponge tissue. Despite the loss of viability in some chambers, the enrichment yielded novel species though very few could be grown as pure cultures. The in situ growth chamber method was the most successful in the isolation of novel species as 40% of the isolates from this method were novel compared to 2% from direct plating (Jung et al. 2021). Therefore, a combination of in situ growth chambers with our plating methods should be very successful in obtaining large numbers of novel species or genera.

Of the 21 identified genera, about half belong to *Actinobacteria*, and the genus *Streptomyces* was dominant, even though no *Streptomyces* were isolated from the Glenelg Block sponges. This location-based lack of a normally predominant genus is difficult to explain, and it cannot be due to the isolation methods which are the same for the Rapid Bay sponges. Similarly, other studies have reported spatial variation in abundance and diversity of bacteria with respect to sample sites and sponge types (Abdelmohsen et al. 2010; Lafi et al. 2005; Thiel et al. 2007; Webster et al. 2001). Some bacterial species were sponge specific and not found in other sponges (Erwin et al. 2011; Schwartz et al. 2014). Spatial variation on the nature of sponges in South Australia marine environments has been documented (Sorokin et al. 2007; Sorokin and Currie 2008). Rapid Bay is further from human habitation compared to Glenelg Blocks which has run-off rain flows as the main factor with more human and animal contact, which could be possible determinants for the variability of bacteria within the sponge communities (Galand et al. 2009; Schmitt et al. 2012).

Our findings predict that marine sponges will continue to be a good source of novel antimicrobials (Anteneh et al. 2021). This is especially the case when similarity analysis of these bacteria with type strains showed about 30% could potentially be new species, as has been found in other studies (Abdelmohsen et al. 2010; Afonso de Menezes et al. 2017; Ahn et al. 2011).

Next-generation sequencing of microbiota from the *A. sulfurea* sponge sample that showed the highest genus diversity revealed the presence of 12 phyla and about 149 genera, with a dominancy of Proteobacteria. Nearly 27% of the genera isolated by culture methods (*Pseudoalteromonas*, *Pseudomonas*, *Pseudonocardia*, *Staphylococcus*, and *Streptomyces*) were shared by NGS*.* Similarly, NGS data from the less diverse *Carteriospongia foliascens* sponge revealed 13 phyla and 192 genera, where about 29% of those genera isolated by culture methods (*Bacillus* and *Streptomyces*) were detected by NGS. In both sponge samples, more than half of the genera isolated were not detected by NGS. Furthermore, NGS data revealed significant variation on diversity of genera within the sponge samples. Similar to our study, several others have reported few shared genera or species using culture-dependent and culture-independent approaches (Jackson et al. 2013; Kisand and Wikner 2003; Knobloch et al. 2019; Stefani et al. 2015; Sun et al. 2010; Yashiro et al. 2011). The findings highlighted that the two approaches are complementary and should be combined to reflect the true picture of a microbial community in the environment. The variety of isolation methods we used in this study helped to report more shared genera among the two approaches.

Here, we can identify several possible factors associated with these variations: (a) primers used for amplification may not amplify all the members in the sponge community, which demands further optimization of the NGS protocol. This is supported by our findings in the previous studies of Yang et al. (2019b) where a variation of phyla and genera is observed among the three primers; (b) the combination of isolation methods employed in this study supports isolation of uncommon genera, which are low in number (for NGS) but readily culturable; (c) while the isolation protocols used should allow the growth of many genera which are known to be culturable, they are still not enough to reflect the total culturable population of bacteria in sponges. This observation reinforces the need for novel isolation protocols.

The diversity of bacterial isolates is highly affected by the types of media used. Our findings indicated the importance of media containing different nutrients for isolation of more diverse bacteria, as demonstrated by isolation of distinct genera on ASP and HV media compared to fewer genera isolated with nutrient poor SWA medium. Furthermore, it showed the specific advantage of one medium type over the other (Bibi et al. 2018; Matobole et al. 2017). Substantial CFU and morphological variations were observed depending on the composition of the medium as well as the presence or absence of enrichment and antibiotics (Schwartz et al. 2014; Selvin et al. 2009; Sipkema et al. 2011).

Prolonged incubation periods were an important facet in this study. In the first few weeks of incubation, isolates were dominated mostly by the genus Bacillus and few other common non-actinobacterial genera. As the incubation period increased so did a shift towards more actinobacterial genera. Incubation time-dependent variation on the abundance and the diversity of bacteria as indicated by Kaewkla and Franco (2013) is once again highlighted in this study, in which about 50% of the isolates were obtained after 6 weeks of incubation. This reinforces the importance of providing an adequate period of incubation to bring out the less common, low abundant bacteria in the culture system.

In general, more non-actinobacterial genera were found under limited oxygen levels and underpin the importance of multiple incubation conditions for isolation of more diverse bacteria from sponge samples. Strict oxygen requirement analysis revealed all the strains isolated from anaerobic conditions grew in the presence of oxygen, which implied the dominancy of facultative anaerobes. The presence of anaerobic bacteria in sponges has been seen before (Mohamed et al. 2010; Schwartz et al. 2014; Selvin et al. 2009; Sipkema et al. 2011). The occurrence of time-based anaerobic patches inside a sponge was indicated, and this condition could arise from the change of water pumping rate or oxygen depletion at night (Hoffmann et al. 2005; 2008).

The tolerance of the bacterial isolates to NaCl indicated that the majority (59%) did not require NaCl, which raises the question whether parts of the internal sponge tissue may be NaCl free. Therefore, all marine-derived bacteria do not appear to have an absolute requirement of salt for growth, though all grew in the presence of 4% (w/v) NaCl and some preferred higher NaCl concentrations (Kennedy et al. 2009).

This study has shown that the majority of bacteria from the marine environment grow within a narrow temperature range of 15 to 27°C, as corroborated by Hicks et al. (2018). Only 1% of the isolates were able to grow between 3 and 37°C, and 20% of all isolates were able to withstand a temperature range of 22–24°C. The narrow temperature tolerance of most of the isolates predicts changes in microbial diversity with changes in the ambient temperature of the seawater and a poor ability to adapt to higher temperatures, such as those associated with climate change.

In conclusion, we have shown the factors that are successful for the isolation of large numbers of diverse bacteria from marine sponge and have described their functional properties.

## Supplementary Information

Below is the link to the electronic supplementary material.

Supplementary file1 (PDF 761 KB)

## Data Availability

All data generated or analyzed during this study are included in this published article (and its Additional files). The raw NGS reads were deposited in the GenBank at the National Center for Biotechnology Information (BioProject ID: PRJNA490791).
