# Supplementary material for: Factors affecting the isolation and diversity of marine sponge-associated bacteria
Source: Appl Microbiol Biotechnol. 2022 Feb 1;106(4):1729–44. doi: 10.1007/s00253-022-11791-8 (PMC8882111; doi:10.1007/s00253-022-11791-8)

Journal Name: APPLIED MICROBIOLOGY AND BIOTECHNOLOGY

Factors affecting the isolation and diversity of marine sponge associated bacteria

Yitayal S Anteneh[1,2,4], Qi Yang[3,4], Melissa H Brown[5] and Christopher MM Franco[1,4 *]

[1]Medical Biotechnology, College of Medicine and Public Health, Flinders University, Bedford Park, SA 5042, Australia

[2]Department of Medical Microbiology, College of Medicine, Addis Ababa University, Addis Ababa, Ethiopia

[3]Center for Marine Drugs, State Key Laboratory of Oncogene and Related Genes, Department of Pharmacy, Renji Hospital, School of Medicine, Shanghai Jiao Tong University, Shanghai 200127, China

[4]Centre for Marine Bioproducts Development, College of Medicine and Public Health, Flinders University, Bedford Park, SA 5042, Australia

[5]College of Science and Engineering, Flinders University, Bedford Park, SA 5042, Australia

*Corresponding author: Christopher Franco (Email:chris.franco@flinders.edu.au;

Tel: +61431500884; Fax: +61872218555)

Table S1 Percentage similarity of 16S rRNA partial gene sequence with nearest type strains.

Isolate	Highest Match	% Sequence Similarity	RFLP groups	Genbank accession number
RB37	*Bacillus algicola KMM 3737T*	99.46	7	MN310361
YA6	*Bacillus algicola KMM 3737T*	99.68	7	MN310362
LC2	*Bacillus flexus* NBRC 15715[T]	98.97	7	MN310326
YA36	*Bacillus haynesii NRRL B-41327T*	99.47	13	MN310336
YAI	*Bacillus hwajinpoensis SW-72T*	99.89	1	MN310359
RB73	*Bacillus hwajinpoensis SW-72T*	99.46	5	MN310357
GB48	*Bacillus hwajinpoensis SW-72T*	100	7	MN315519
LC16	*Bacillus hwajinpoensis SW-72T*	99.68	10	MN310328
GB3	*Bacillus hwajinpoensis SW-72T*	100	36	MN315520
RB138	*Bacillus infantis NRRL B-14911*	99.46	7	MN310331
D5	*Bacillus licheniformis ATCC 14580T*	99.15	8	MN310334
LC35	*Bacillus pumilus* ATCC 7061[T]	98.44	35	MN310333
GB21	*Bacillus qingshengii* G19[T]	98.25	1	MN310332
RB62	*Bacillus siamensis KCTC 13613T*	99.14	22	MN310330
LC30	*Bacillus simplex NBRC 15720T*	99.48	5	MN310327
RB99	*Bacillus simplex NBRC 15720T*	99.87	7	MN310354
RB148	*Bacillus simplex NBRC 15720T*	99.3	7	MN310352
RB14	*Bacillus sonorensis NBRC 101234T*	99.36	13	MN310358
LC15	*Falsibacillus pallidus CW 7T*	99.12	10	MN310329
LC7	*Fictibacillus rigui WPCB074T*	99.48	9	MN310335
GB17	*Gordonia otitidis* NBRC 100426[T]	99.1	15	MN310343
GB17[a]	*Gordonia otitidis* NBRC 100426[T]	98.89	15	MN310343
L40	*Isoptericola rhizophila* BKS 3-46[T]	97.87	19	MN310363
L40[a]	*Isoptericola rhizophila* BKS 3-46[T]	98.3	19	MN315548
L3	*Isoptericola rhizophila* BKS 3-46[T]	97.87	19	MN315523
F12	*Isoptericola rhizophila* BKS 3-46[T]	97.87	19	MN315524
GB23	*Janibacter indicus CGMCC 1.12511T*	99.79	1	MN310348
RB40	*Kocuria kristinae NBRC 15354T*	99.45	21	MN310355
RB107	*Kocuria kristinae NBRC 15354T*	99.45	34	MN310353
RB86	*Leisingera methylohalidivorans* DSM 14336[T]	99.10	16	MN310320

GB11	*Limimaricola hongkongensis* DSM 17492^T	98.77	4	MN315530
GB46	*Limimaricola hongkongensis* DSM 17492^T	98.95	6	MN310319
GB33	*Limimaricola hongkongensis* DSM 17492^T	98.56	6	MN315531
A4	*Microbacterium aquimaris* JS54-2^T	99.35	20	MN315525
YA34	*Microbacterium aquimaris* JS54-2^T	98.74	26	MN310337
RB5	*Micrococcus aloeverae* AE-6^T	99.60	23	MN310360
LC12	*Micrococcus aloeverae* AE-6^T	99.60	24	MN310338
RB166	*Micrococcus aloeverae* AE-6^T	99.43	29	MN315526
GB37	*Muricauda aquimarina* SW-63^T	97.67	3	MN310365
GB37[a]	*Muricauda aquimarina* SW-63^T	98.13	3	MK367393
LC1	*Mycolicibacterium iranicum* DSM 45541^T	98.68	17	MN310364
LC1[a]	*Mycolicibacterium iranicum* DSM 45541^T	98	17	MK358953
RB122	*Pseudoalteromonas marina* Mano4^T	99.28	25	MN310325
LC17	*Pseudomonas oleovorans subsp. oleovorans* DSM 1045^T	100	14	MN315527
YA11	*Pseudonocardia carboxydivorans* Y8^T	99.74	30	MN310349
LC21	*Rhodococcus corynebacterioides* DSM 20151^T	99.06	12	MN310339
GB15	*Rhodovulum iodosum* N1^T	98.09	11	MN310321
RB71	*Rothia kristinae* NBRC 15354^T	99.86	33	MN310345
L11	*Staphylococcus hominis subsp. novobiosepticus* GTC 1228^T	100	18	MN315522
RB38	*Streptomyces ambofaciens* ATCC 23877^T	98.60	32	MN310342
YA19	*Streptomyces anthocyanicus* NBRC 14892^T	98.55	31	MN310340
H8	*Streptomyces antimycoticus* NBRC 12839^T	100	38	MN310344
YA20	*Streptomyces bacillaris* NBRC 13487^T	98.87	31	MN315528
YA27	*Streptomyces badius* NRRL B-2567^T	99.84	31	MN315521
RB128	*Streptomyces hyderabadensis* OU-40^T	99.44	31	MN310350
RB27	*Streptomyces lienomycini LMG 20091T*	99.83	32	MN315546
RB27a	*Streptomyces lienomycini LMG 20091T*	99.92	32	MN315546
YA22	*Streptomyces prasinosporus NRRL B-12431T*	98.40	31	MN310366
YA22a	*Streptomyces prasinosporus NRRL B-12431T*	98.94	31	MN315547
LC14	*Streptomyces setonii NRRL ISP-5322T*	99.85	37	MN310347
RB154	*Streptomyces sundarbansensis MS1/7T*	98.30	31	MN310351
YA2	*Streptomyces tendae ATCC 19812T*	99.82	31	MN310341

RB56	*Streptomyces violaceorubidus LMG 20319T*	99.65	31	MN310346
RB58	*Sulfitobacter delicates DSM 16477T*	99.89	2	MN310356
GB12	*Sulfitobacter faviae S5-53T*	99.30	1	MN315529
GB44	*Sulfitobacter faviae S5-53T*	99.27	2	MN310324
GB39	*Sulfitobacter indolifex HEL-45T*	98.87	27	MN310322
GB6	*Sulfitobacter indolifex HEL-45T*	99.20	28	MN310323

Table S2 Genus diversity with respect to isolation temperature

Genera	15°C	27°C	Genera	15°C	27°C
Gordonia	2	5	*Bacillus*	30	61
Isoptericola	1	4	*Fictibacillus*	0	7
Janibacter	0	1	*Flasibacillus*	0	6
Kocuria	11	20	*Staphylococcus*	0	5
Microbacterium	0	17	*Leisingera*	2	3
Micrococcus	7	8	*Limimaricola*	8	9
Mycolicibacterium	0	3	*Pseudoalteromonas*	0	1
Pseudonocardia	0	7	*Pseudomonas*	4	2
Rhodococcus	2	6	*Rhodovulum*	2	3
Streptomyces	25	86	*Sulfitobacter*	13	19
Muricauda	0	3			

Table S3 List of Phyla identified by Next Generation Sequencing from sponges *Aplysilla sulfurea* and *Carteriospongia foliascens.*

Aplysilla sulfurea (n=12)	*Carteriospongia foliascens* (n=13)
Actinobacteria	*Actinobacteria*
Bacteroidetes	*Bacteroidetes*
Chlamydiae	*Chlamydiae*
Chloroflexi	*Crenarchaeota*
Crenarchaeota	*Cyanobacteria*
Cyanobacteria	*Euryarchaeota*
Firmicutes	*Firmicutes*
Fusobacteria	*Fusobacteria*
Planctomycetes	*Planctomycetes*
Proteobacteria	*Proteobacteria*
Spirochaetes	*Spirochaetes*
Verrucomicrobia	*Tenericutes*
	Verrucomicrobia

Table S4 Known and candidate genera identified by Next Generation Sequencing from sponge *Aplysilla sulfurea.*

137 known genera identified by NGS			
Acidaminobacter	*Desulfovibrio*	*Parachlamydia*	*Thermoanaerobacter*
Acinetobacter	*Dialister*	*Paracoccus*	*Treponema*
Actinobacillus	*Dinoroseobacter*	*Persicirhabdus*	*Turicibacter*
Actinomyces	*Dolichospermum*	*Persicobacter*	*Ulvibacter*
Aggregatibacter	*Elizabethkingia*	*Phaeobacter*	*Veillonella*
Agrobacterium	*Erwinia*	*Photobacterium*	*Verrucomicrobium*
Alkaliphilus	*Erythrobacter*	*Planctomyces*	*Vibrio*
Alteromonas	*Ferrimonas*	*Pleomorphomonas*	*Waddlia*
Amaricoccus	*Flavobacterium*	*Plesiomonas*	*Winogradskyella*
Aminobacter	*Fluviicola*	*Polaribacter*	*Wolbachia*
Amycolatopsis	*Fusibacter*	*Porphyromonas*	*Zoogloea*
Antarctobacter	*Fusobacterium*	*Prevotella*	*Candidatus Xiphinematobacter*
Aquicella	*Gemella*	*Prochlorococcus*	*Capnocytophaga*
Aquimarina	*Glaciecola*	*Propionibacterium*	*Chryseobacterium*
Arcobacter	*Gramella*	*Pseudaminobacter*	*Clostridium*
Atopobium	*Granulicatella*	*Pseudidiomarina*	*Cobetia*
Bacteroides	*Haemophilus*	*Pseudoalteromonas*	*Cohaesibacter*
Bdellovibrio	*Inquilinus*	*Pseudomonas*	*Collinsella*
Bifidobacterium	*Jannaschia*	*Pseudonocardia*	*Congregibacter*
Burkholderia	*Kaistia*	*Psychrilyobacter*	*Coprococcus*
Campylobacter	*Kingella*	*Reinekea*	*Coraliomargarita*
Candidatus Arthromitus	*Klebsiella*	*Rhodobacter*	*Corynebacterium*
Candidatus Portiera	*Lactobacillus*	*Robiginitalea*	*Coxiella*
Candidatus Protochlamydia	*Leptotrichia*	*Roseivirga*	*Crenothrix*

Alkaliphilus	*Erythrobacter*	*Planctomyces*	*Vibrio*
Candidatus Rhabdochlamydia	*Leuconostoc*	*Roseobacter*	*Desulfobacter*
Desulfocapsa	*Loktanella*	*Roseovarius*	*Moritella*
Desulfosarcina	*Luteolibacter*	*Rothia*	*Nautella*
Desulfosporosinus	*Lutimonas*	*Rubritalea*	*Neptunomonas*
Maribacter	*Ruegeria*	*Microbulbifer*	*Nitrosopumilus*
Marivita	*Selenomonas*	*Micrococcus*	*Octadecabacter*
Megasphaera	*Shewanella*	*Moraxella*	*Oleibacter*
Oribacterium	*Staphylococcus*	*Streptococcus*	*Sutterella*
Parabacteroides	*Stenotrophomonas*	*Streptomyces*	*Synechococcus*
Syntrophomonas	*Tepidibacter*	*Thalassobius*	*Thalassomonas*
Tenacibaculum			
12 candidate genera identified by NGS			
A1	*HB2-32-21*	*[Prevotella]*	*WH1-8*
BD2-13	*HTCC*	*T78*	*ZD0117*
Blvii28	*MSBL3*	*Ucs1325*	*5-7N15*

Table S5 Known and candidate genera identified by Next Generation Sequencing from sponge *Carteriospongia foliascens*

183 known genera identified by NGS				
Acaryochloris	*Cetobacterium*	*Haemophilus*	*Odoribacter*	*Roseivivax*
Acholeplasma	*Clostridiisalibacter*	*Haloplanus*	*Oleibacter*	*Roseobacter*
Achromobacter	*Clostridium*	*Halorhabdus*	*Oscillospira*	*Roseovarius*
Actinobacillus	*Cohaesibacter*	*Hyphomicrobium*	*Paenibacillus*	*Rothia*
Actinomyces	*Collinsella*	*Hyphomonas*	*Parabacteroides*	*Rubritalea*
Adlercreutzia	*Congregibacter*	*Idiomarina*	*Parachlamydia*	*Ruegeria*
Akkermansia	*Coprococcus*	*Inquilinus*	*Paracoccus*	*Ruminococcus*
Alcanivorax	*Coraliomargarita*	*Janibacter*	*Paulinella*	*Rummeliibacillus*
Aliivibrio	*Corynebacterium*	*Jannaschia*	*Persicirhabdus*	*Saprospira*
Alkaliphilus	*Coxiella*	*Jeotgalicoccus*	*Persicobacter*	*Shewanella*
Allobaculum	*Dehalobacterium*	*Klebsiella*	*Phaeobacter*	*Spongiibacter*
Alteromonas	*Demequina*	*Lactobacillus*	*Photobacterium*	*Staphylococcus*
Amaricoccus	*Desulfarculus*	*Lactococcus*	*Piscirickettsia*	*Stenotrophomona s*
Aminobacter	*Desulfobacter*	*Legionella*	*Planctomyces*	*Streptococcus*
Amphritea	*Desulfococcus*	*Leptolyngbya*	*Pleomorphomonas*	*Streptomyces*
Amycolatopsis	*Desulfofaba*	*Leptotrichia*	*Pleomorphomonas*	*Psychrilyobacter*
Anaerococcus	*Desulfomonile*	*Leuconostoc*	*Polaribacter*	*Succinivibrio*
Anaeroplasma	*Desulforhopalus*	*Loktanella*	*Prevotella*	*Succinivibrio*
Anaerospora	*Desulfotalea*	*Luteolibacter*	*Prochlorococcus*	*Synechococcus*
Aquicella	*Desulfovibrio*	*Lutibacterium*	*Propionibacterium*	*Syntrophomonas*
Arcobacter	*Devosia*	*Lutimonas*	*Propionigenium*	*Tatlockia*
Arthrobacter	*Dinoroseobacter*	*Marinicella*	*Pseudaminobacter*	*Tepidibacter*
Atopobium	*Dolichospermum*	*Marinobacter*	*Pseudoalteromonas*	*Tepidimonas*
Azospirillum	*Dorea*	*Marinomonas*	*Pseudomonas*	*Thalassobius*
Bacillus	*Elizabethkingia*	*Marivita*	*Pseudonocardia*	*Thalassomonas*

Bacteroides	*Enterobacter*	*Methylobacterium*	*Pseudoruegeria*	*Thiohalorhabdus*
Balneola	*Enterococcus*	*Microbulbifer*	*Psychrilyobacter*	*Tolumonas*
Bdellovibrio	*Erwinia*	*Moraxella*	*Psychrobacter*	*Trichococcus*
Bifidobacterium	*Faecalibacterium*	*Moritella*	*Psychromonas*	*Trichodesmium*
Blautia	*Ferrimonas*	*MSBL3*	*Ralstonia*	*Turicibacter*
Burkholderia	*Flavobacterium*	*Nautella*	*Reinekea*	*Veillonella*
Campylobacter	*Fluviicola*	*Neisseria*	*Rhizobium*	*Verrucomicrobiu m*
Candidatus Hepatoplasma	*Gemella*	*Nitratireductor*	*Rhodovulum*	*Vibrio*
Candidatus Portiera	*Geobacter*	*Nitrosopumilus*	*Rickettsiella*	*Waddlia*
Candidatus Protochlamydia	*Gloeobacter*	*Nitrospina*	*Robiginitalea*	*Winogradskyella*
Candidatus Rhabdochlamydia	*Gluconacetobacter*	*Oceanospirillum*	*Roseivirga*	*Wolbachia*
Candidatus Xiphinematobacter	*Granulicatella*	*Octadecabacter*		
9 candidate genera identified by NGS				
5-7N15	*A17*	*B46*	*heteroC45*	*HTCC*
SJA-88	*SJA-88*	*Ucs1325*	*Ucs1325*	

Figure S1: Twelve sponge samples collected from two sites. RB 1, RB 2, RB 3, RB 11, RB 12, RB 16, RB 17, and RB 18 were collected from Rapid Bay; GB 1, GB 08, GB 21, and GB 23 were collected from Glenelg Blocks.

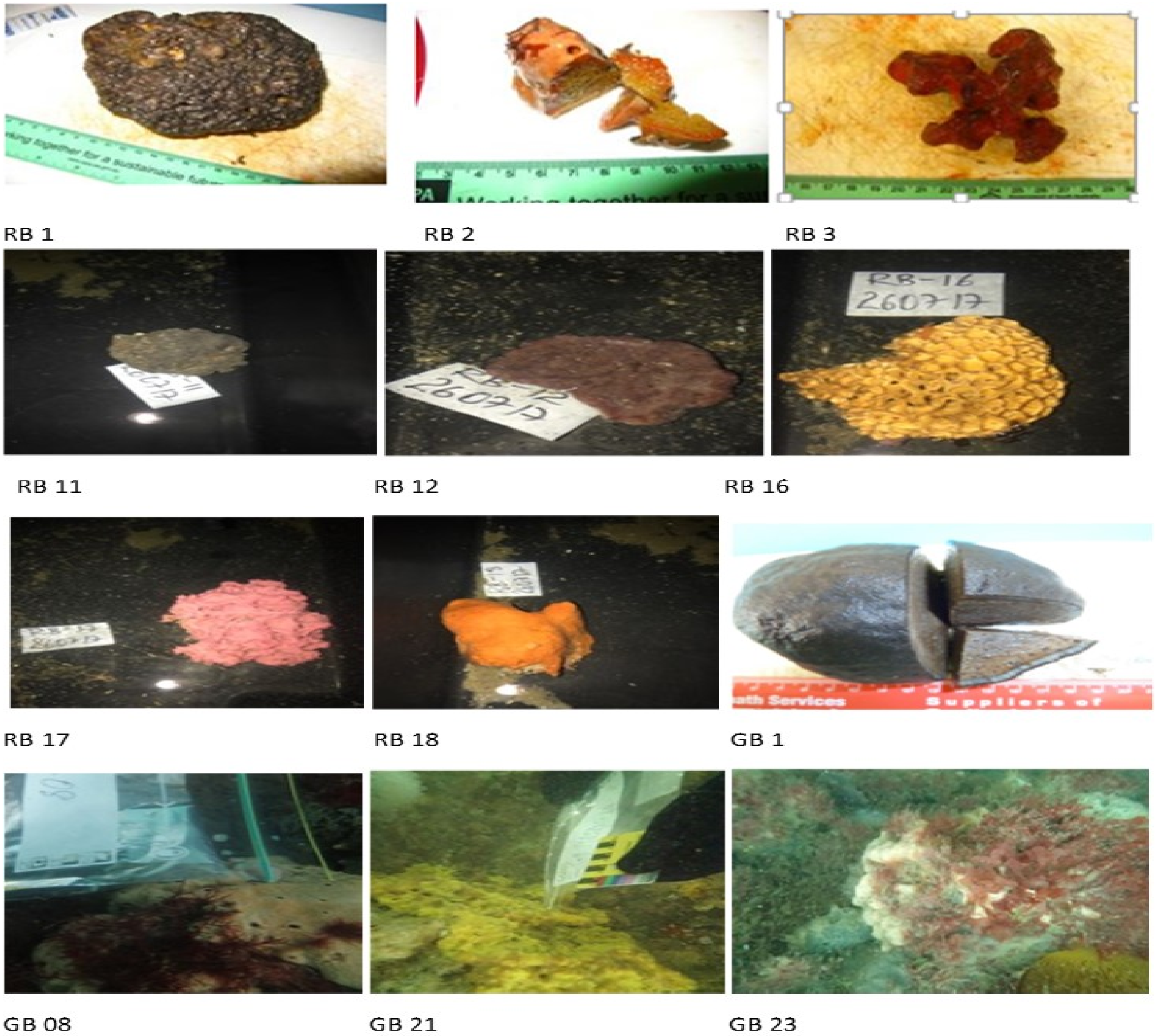

Figure S2: Cumulative abundance of isolates collected over the 16 weeks of incubation.

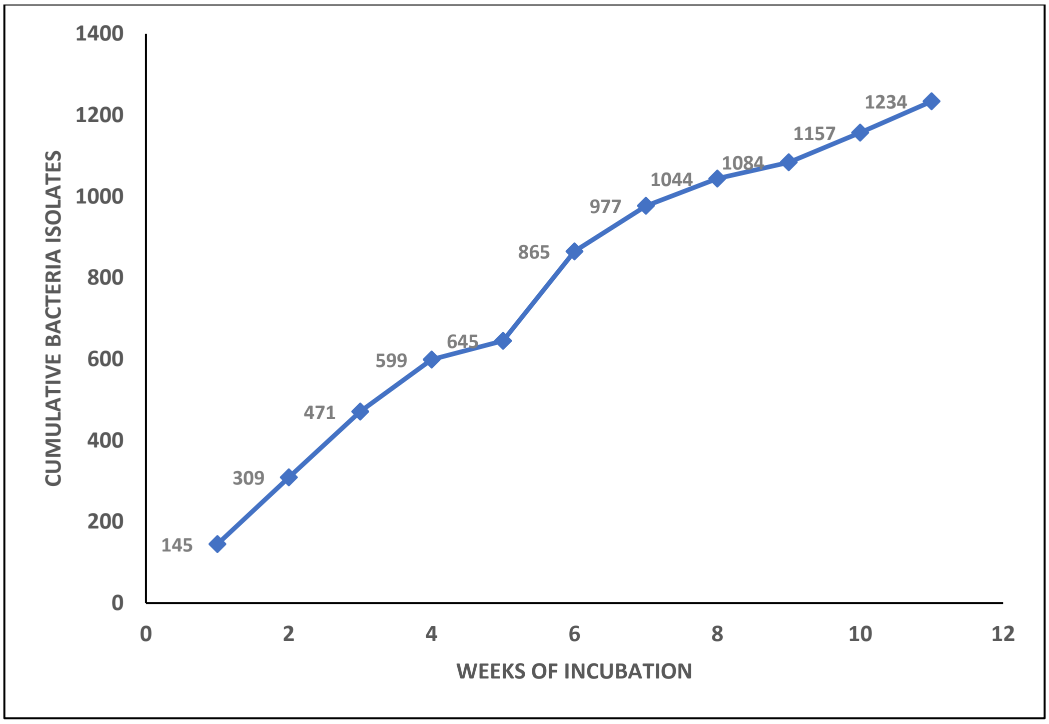

Figure S3 Abundance and diversity of bacteria isolated under different incubation conditions.

a) total colony forming bacteria and morphological forms; **b)** the diversity of genera isolated at different oxygen levels

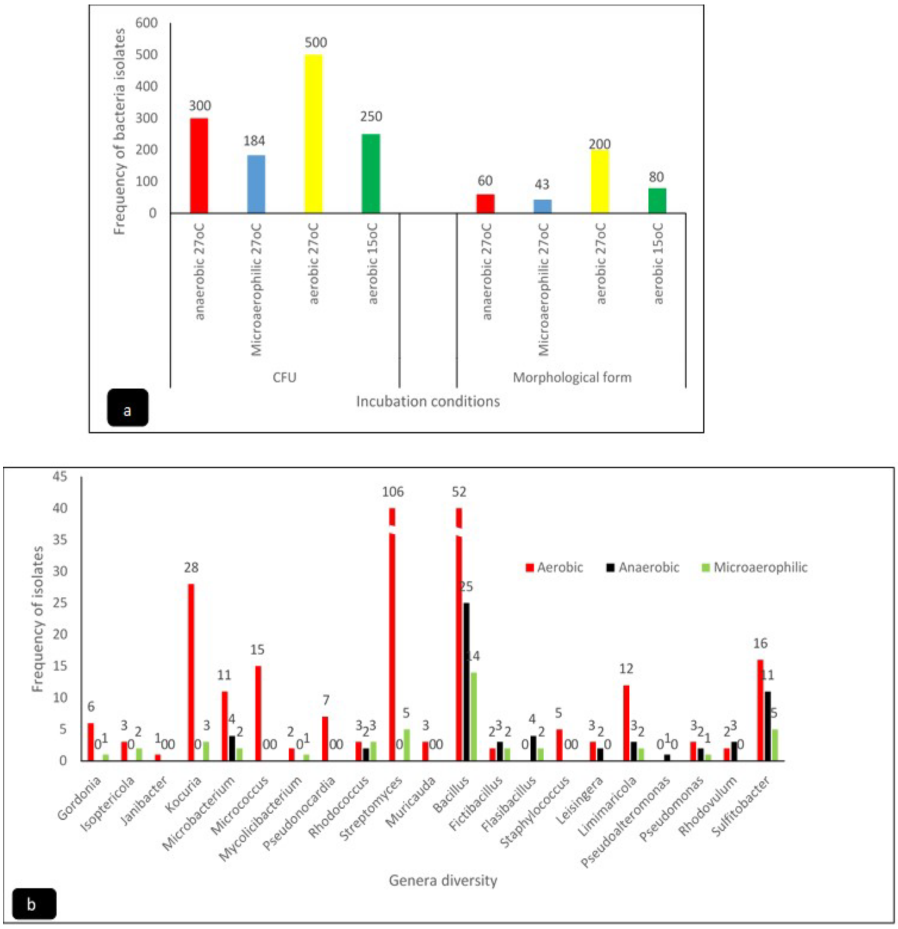

Figure S4: Bacterial growth at different NaCl concentrations presented as a percentage of total number.

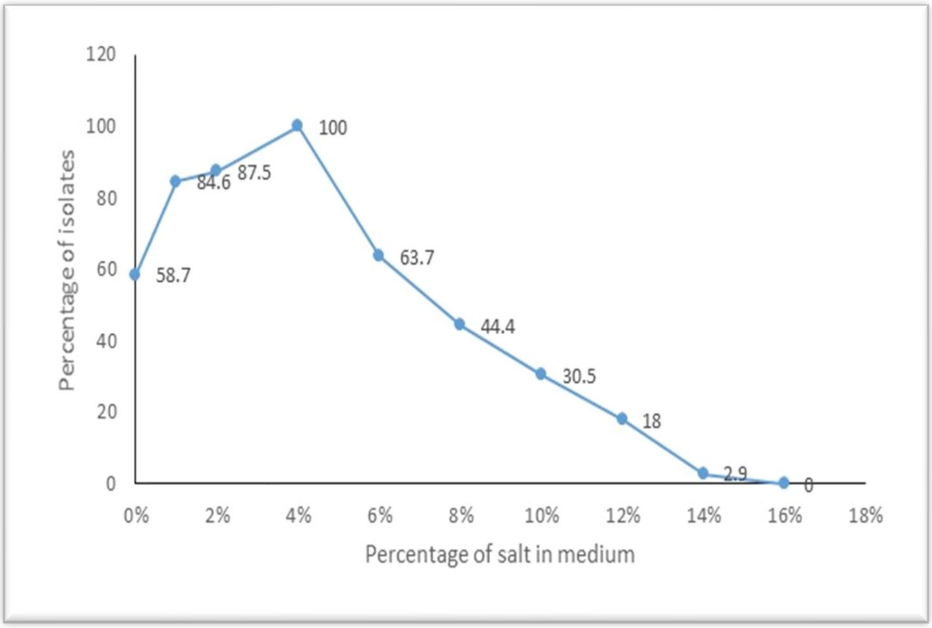

Supplement: Supplementary file 1 — Supplementary file1 (PDF 761 KB) [file 253_2022_11791_MOESM1_ESM.pdf]
